# Salivary Cortisol Detection with a Fully Inkjet-Printed Paper-Based Electrochemical Sensor

**DOI:** 10.3390/mi15101252

**Published:** 2024-10-12

**Authors:** Miguel Zea, Hamdi Ben Halima, Rosa Villa, Imad Abrao Nemeir, Nadia Zine, Abdelhamid Errachid, Gemma Gabriel

**Affiliations:** 1 Instituto de Microelectrónica de Barcelona IMB-CNM (CSIC), Campus Universitat Autònoma de Barcelona, 08193 Cerdanyola del Vallès, Spain; miguezea@hotmail.com (M.Z.); rosa.villa@imb-cnm.csic.es (R.V.); gemma.gabriel@imb-cnm.csic.es (G.G.); 2 Institut UTINAM, UMR CNRS 6213, Université de Franche-Comté, 16 Route de Gray, 25030 Besançon, France; 3 CIBER de Bioingeniería, Biomateriales y Nanomedicina (CIBER-BBN), 08193 Bellaterra, Spain; 4 ThEA Group, Department of Chemistry and Biochemistry, Faculty of Arts and Science, Holy Spirit University of Kaslik (USEK), Jounieh P.O. Box 446, Lebanon; imad-nmeir@hotmail.com; 5 Institut des Sciences Analytiques (ISA), Université Claude Bernard Lyon 1, 5 Rue de la Doua, 69100 Lyon, France; nadia.zine@univ-lyon1.fr (N.Z.); abdelhamid.errachid-el-salhi@univ-lyon1.fr (A.E.)

**Keywords:** inkjet printing, paper substrate, cortisol, stress hormone, electrochemical impedance spectroscopy, electrochemical sensor

## Abstract

Electrochemical paper-based analytical devices (ePADs) offer an innovative, low-cost, and environmentally friendly approach for real-time diagnostics. In this study, we developed a functional all-inkjet paper-based electrochemical immunosensor using gold (Au) printed ink to detect salivary cortisol. Covalent binding of the cortisol monoclonal antibody onto the printed Au surface was achieved through electrodeposition of 4-carboxymethylaniline (CMA), with ethanolamine passivation to prevent non-specific binding. The ePAD exhibited a linear response within the physiological cortisol range (5–20 ng/mL), with sensitivities of 25, 23, and 19 Ω·ng/mL and R^2^ values of 0.995, 0.979, and 0.99, respectively. Additionally, interference studies against tumor necrosis factor-α (TNF-α) and N-terminal pro-B-type natriuretic peptide (NT-proBNP) yielded excellent results. This novel ePAD, fabricated using inkjet printing technology on paper, simplifies the process, reduces environmental impact, and lowers fabrication costs.

## 1. Introduction

One of the most common materials used for sensor development for bioanalytical samples detection is paper. Usually referred as paper-based analytical devices (PADs) or micro-paper-based analytical devices (µPADs), they are an attractive alternative due to their 3D fibrous structure, inertness to generally utilized reagents, chemical stability, biocompatibility, biodegradability, availability, their light weight, ease of production, and modification [1]. PADs or µPADs are made of an arrangement of hydrophilic channels combined with hydrophobic structures in a simple pattern that is either printed or cut on a paper with specialized technologies [2], thus, endowing the fabricated device with a pump-free microfluidic system [3,4,5].

The latest trends in the literature favors paper substrates over other materials for developing eco-friendly, inexpensive, and non-reusable sensing devices [6,7].

Electrochemical PADs and µPADs (ePADs) are lauded as game changers when it comes to the development of efficient point-of-care devices due to their potential in creating devices that are simpler and more reliable than colorimetric or single-electrode sensors without compromising high sensitivity, with the added benefit of being easily disposable [8].

ePADs use as sensors have exploded to include the chemical, environmental, food processing, biomedical, and clinical analysis industries, to name but a few [9]. Electrochemical sensors are low cost, have high selectivity and sensitivity, can perform a variety of assays, and can utilize a wide variety of miniaturized detection techniques [10]. Therefore, ePADs role is to further miniaturize the electrodes by varying their deposition on their substrate using a number of recently developed technologies [7]. Amongst these methods, screen-printing stands above most techniques as the most utilized for the production of electrodes on ePADs [1]. While several roadblocks remain, and innovation is crucial to keep pace with modern demands, the search for a great leap forward in the field of ePAD fabrication techniques continues, with inkjet printing technology showing the greatest promise [11].

The inkjet printing technology ease of layout modifications, without screen or stencils use, its waste reduction, and its non-contact deposition provide it with an edge over traditional ePAD fabrication techniques [12]. In addition, inkjet printing’s non-contact deposition provides a contamination resistant of the substrate while remaining compatible with delicate and super-thin paper substrates that are compromised by force application with other techniques. Thus, inkjet printing technology for paper-based devices is forging forth a path for ePADs to be used as biological and chemical sensors [13]. Despite these advantages, inkjet-printed ePADs are mostly used for the measurement and detection of simple molecules without intensive electrode modification [13,14,15,16,17].

Our research’s aim is to detect cortisol with an inkjet-printed-based ePAD on a physiological fluid, saliva, in order to demonstrate inkjet-printed electrode on paper’s capability to behave as a traditional electrode by bio-functionalizing it with anti-cortisol antibodies for direct detection of cortisol.

Cortisol hormone is an important biomarker to measure animals and human levels of stress, thus, earning the moniker of “the stress hormone”. Cortisol’s roles in the body include, but are not limited to, regulating blood pressure, the immune system, protein and carbohydrate cellular intake, adipose metabolism, and anti-inflammatory action. Cortisol sensors development is an active field of study because it plays an active role in the management of diseases such as heart failure, Adison’s disease, Cushing syndrome, and even stress levels [18].

Electrochemical measurements have been used successfully over the past decade to detect cortisol in an assortment of biological matrices such as hair, sweat, saliva, serum, blood, and plasma, with the added benefit that electrochemical measurements are constantly being integrated into portable and wearable devices for the fast and precise detection of cortisol [19,20,21]. Various transducers exist to provide the developed sensor with its specificity, such as quantitative biomarkers, which have been employed to electrochemical sensors and imagery techniques [22,23,24]. However, immunoassays or immunosensing techniques and sensing receptors remain at the forefront in screening methods innovation [25]. The antigen/antibody binding is the most reliable method for portable and disposable cortisol detection devices owing to its wide employment [19,20,26,27]. On the one hand, image analysis methods necessitate bulky optical detectors and complex equipment. In contrast, electrochemical sensors have been miniaturized to the point of successfully integrating them into wearable, portable, point-of-care devices. The sensor’s surface must be modified with the proper transducer to interact and register exclusively cortisol molecules. This will, in turn, induce a change in the transducer’s electrical properties, which are then picked up and recorded as an electrochemical signal, thus acting as a cortisol electrochemical sensor.

As far as our knowledge extends, an inkjet-printed cortisol electrochemical sensor technology that detects in a physiological fluid is yet to be reported in the literature. As such, we present the first ever fully printed biosensor on paper that detects cortisol in the physiological range in untreated human saliva, paving the way for further development of inkjet-printed paper-based biosensing systems.

The platform we developed is made up of four gold (Au) working microelectrodes (WEs), two Au counter microelectrodes (CEs), and two pseudo-references (REs), conferring the capacity for simultaneous detection, which grants us greater sensitivity and ease for repeatability of our developed sensor for upward of *n* = 4 samples. The WEs were functionalized with diazonium salt, then cortisol antibodies were immobilized on them with EDC/NHS. The technique used for the detection was electrochemical impedance spectroscopy (EIS). Our innovative and novel approach with an inkjet-printed paper-based biosensor for cortisol detection is a very attractive proposition as it establishes the first steps in using inkjet printing technology for the development of platforms for the detection of hormones and cytokines.

## 2. Materials and Methods

### 2.1. Materials and Chemicals

For the development of the printed platform, we used three commercially available inks: A silver (Ag) nanoparticle ink (Dupont-PE410, Auburn Hills, MI, USA), SU-8 ink (2002 from MicroChem, Newton, MA, USA), and a low-curing Au colloidal ink (DryCure Au-J 1010B from Colloidal Ink Co., Ltd., Okayama, Japan). Additionally, we developed a silane-based ink [28] for substrate treatment using 1H,1H,2H,2H-perfluorooctyltriethoxysilane (POTS, from abcr GmbH, Karlsruhe, Germany), Tetraethyl orthosilicate 98+% (TEOS), hydrochloric acid 36% (HCl), absolute ethanol, and distilled water. The printer used for all the ink formulations is a drop-on-demand Dimatix Materials Printer (DMP 2831 from Fujifilm Dimatix, Santa Clara, CA, USA). For testing the pseudo-reference electrode (pRE), the printed Ag electrode was chlorinated with HCl (0.1 M) and potassium chloride (KCl, 0.1 M). The substrate used is Whatman cellulose paper grade 1 (WHA3001861). Its properties are 180 μm thickness, a porosity of 0.686, and pore diameter of 11 µm.

To activate and characterize the printed microelectrodes, potassium hexacyanoferrate (III) (K_3_[Fe(CN)_6_]), potassium hexacyanoferrate (II) trihydrate (K_4_[Fe(CN)_6_]), and phosphate-buffered saline (PBS) were used. For the biofunctionalization, sodium chloride (NaCl), hydrochloride (EDC-HCl), sodium nitrate (NaNO_2_), ethanolamine (from Fluka, Saint Aubin, France), 4-carboxymethylaniline (CMA), N-hydroxysuccinimide (NHS), and N-(3-dimethylaminopropyl)-N’-ethylcarbodiimide were used. Artificial saliva was fabricated with urea, mucin (from pork stomach extract, type II), calcium chloride (CaCl_2_, purity ≥ 93%), sodium chloride (NaCl, purity ≥ 99.5%), potassium chloride (KCl, purity 99–100.5%), and sodium phosphate dibasic (NaHPO_4_, PharmaGrade). Except for those explicitly mentioned, all reagents were purchased from Merck KGaA (Darmstadt, Germany) and were used without further purification.

The external reference Ag/AgCl electrode and the counter platinum electrode were procured from BVT Technologies (Strážek, Czech Republic). EIS measurements were carried out using a PalmSens4 potentiostat purchased from De Indruk (Ede, The Netherlands). The modeling of the obtained EIS data was achieved using the PSTRACE 5.7 software.

The antibodies used are anti-cortisol monoclonal antibody (Cat. No. XM210) from Novus Biological (Seiche, France). It was purchased alongside human TNF-α antigens (TNF-α) (Car. No. 210-TA), which was purchased from R&D Systems (Lille, France), hydrocortisone (cortisol, Cat. No. ab141250) from Abcam (Paris, France), and NT-proBNP (Cat. No. 8NT2) from HyTest (Torku, Finland).

### 2.2. Preparation of Aliquots

A stock solution of 1000 μg/mL of cortisol was prepared by dissolving the appropriate amount of the pure compound in pure ethanol. Cortisol working and standard solutions at different concentration (10 μg/mL, 100, 20, 15, 10, and 5 ng/mL) were subsequently prepared by diluting the stock solution with PBS. Anti-cortisol (5 µg/mL) has been prepared similarly.

Standard solutions containing other heart failure (HF) biomarkers in PBS (e.g., NT-proBNP and TNF-α in the concentration range 5–20 ng/mL) have been prepared similarly to carry out the interference study.

For the standard solutions of antibody and antigens, the same protocol was used but in the artificial saliva with the same concentration range as were previously prepared. The formulation of artificial saliva followed the protocols found in these articles [29] and consist of dissolving 0.6 g/L of anhydrous calcium chloride (CaCl_2_), 0.4 g/L of sodium chloride (NaCl), 4 g/L urea, 0.6 g/L of sodium phosphate dibasic (Na_2_HPO_4_), 0.4 g/L of potassium chloride (KCl), and 4 g/L mucin in deionized water. The pH is then adjusted to pH 7.2 by adding NaOH or HCl (both 0.1 M) when needed. The solution is then sterilized by autoclaving and stored at −4 °C until use.

### 2.3. Fabrication of Paper-Based Inkjet Electrode

**Figure 1:**
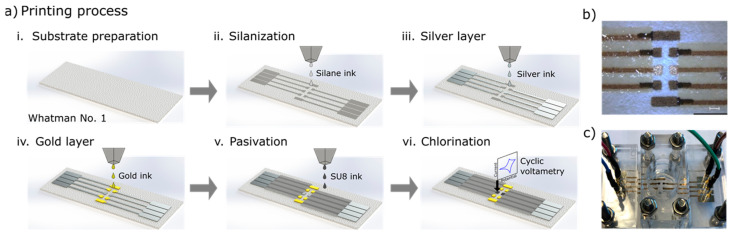
(**a**) Illustration of the microelectrode printing process: (**i**) substrate preparation; (**ii**) printing silane ink for turning the substrate hydrophobic; (**iii**) printing of the Ag pRE, connections and pads followed by a drying step; (**iv**) printing Au ink WEs, followed by a sintering step; (**v**) printing of the passivation SU-8 layer, followed by soft bake and UV curing; (**vi**) chlorination by CV of silver elements to form the pRE-electrode. (**b**) Close-up picture of the microelectrodes showing the 4 WE, 2 pRE, and 2 CE. (**c**) Picture of the microelectrodes inside an electrochemical cell.

We first followed our previously reported protocol to fabricate the paper-based inkjet-printed microelectrodes [30]. The structure of the microelectrode was designed using CleWin 5 software before exporting it to bitmap file format (.bmp) and loading into the Dimatix Bitmap editor. The electrode printing was carried out with a 10-pL printhead in standard laboratory condition, but without particle filtering, or temperature or humidity control. The end result is a complete electrochemical cell that is made up of four working electrodes (WE), two references (RE), and two counter electrodes (CE), as seen in Figure 1b.

The printing process for the microelectrode on paper substrate is illustrated in Figure 1a. First, to create a hydrophobic surface for the electrodes, silane ink was printed (a.ii). This was followed by printing a layer of Ag ink for conductivity elements such as track and pads, and to create the RE (500 × 500 µm) with a drop spacing (DS) of 20 µm (a.iii). The silver elements were then fried at 80 °C for 15 min. The fourth step was printing the gold elements, which are the four Au WEs 800 × 800 µm and the two Au CEs 800 × 3000 µm with a DS of 15 µm (a.iv). The Au ink was then fried before the entire electrode was sintered in the oven at 140 °C for 60 min. To define the active areas of the microelectrode and the pads used to connect them, the areas of the Ag tracks that needed isolation were printed over with the dielectric ink SU-8 (a.v) with a DS of 15 µm. This ink was first cured with a soft bake on a hot plate at 100 °C for 5 min, then it was treated under UV light for 30 s to polymerize the layer with polymer cross-linking. The final result can be seen in Figure 1b.

The final step was the activation of the electrodes, which was achieved using two electrochemical processes. First, the Ag RE was chlorinated (a.vi) using cyclic voltammetry (CV) (Figure S1) in 0.1 M HCl, the scanning the potential window was 0 to 0.2 V versus a commercial Ag/AgCl RE at 20 mV/s. This results in a stable Ag/AgCl pRE. Then, the Au WE were produced by amperometric pulses (5 pulses for 5 s from 0 to −0.2 V) in PBS [31].

### 2.4. Optimization of CMA Electrodeposition

To optimize the functioning of the biosensor, we electrodeposited CMA 5 mM solution on the printed Au WEs with the set-up seen in Figure 1c. We then varied the CV speeds (25, 50, and 80 mV/s) and the number of cycles (5, 6, and 7). The results of this electrodeposition were then assessed using CV in ferro/ferricyanide 10 mM, the speed of scan is 25 mV/s from −0.2 to 0.5 V.

### 2.5. Biofunctionalization of Au WE

Biofunctionalization was developed and optimized in previous works, found here [30].

**Figure 2:**
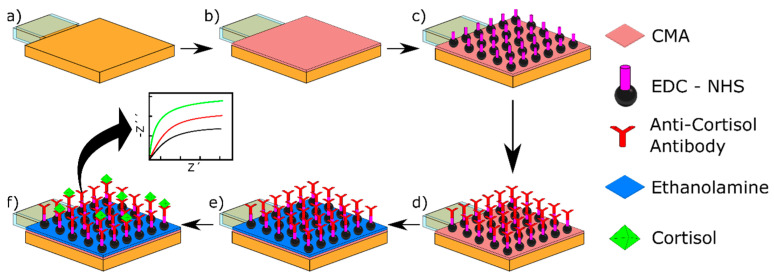
Schematic recreation of the bio-functionalization of a WE (**a**) representation of an activated gold electrode, (**b**) electrodeposition of CMA on gold WEs by applying CV, (**c**) CMA activation by incubation of mixture EDC/NHS, (**d**) bio-functionalization through incubation with anti-cortisol antibodies, (**e**) deactivation of the remaining active groups by incubation in ethanolamine solution, (**f**) incubation of the platform into cortisol solution followed by detection of cortisol by EIS.

Following the activation of the WEs as described above (Figure 2a), the WEs were characterized with CV in CV in ferro/ferricyanide (10 mM) solution. CMA was then electrodeposited, with CV, on the WEs in order to coat them with –COOH functional groups [29]. This was achieved by first mixing 5 mM CMA with 15 mM HCl and 15 mM NaNO_2_ before refrigerating the mixture at 4 °C for 2 h. The electrodeposition was then carried out by CV with the parameters of seven cycles, 25 mV/s, from −1.2 to 0.2 V. This ensured the electrodeposition of a fully blocking layer of CMA on the Au WE (Figure 2b). Following CMA electrodeposition, the electrodes were rinsed with water and dried with nitrogen.

To functionalize the electrode with the anti-cortisol antibodies, first, the CMA groups on the electrode were activated using EDC/NHS (0.4 M/0.1 M) in ethanol for 1 h at room temperature (Figure 2c), then rinsed with HCl 0.1 M to remove EDC/NHS excess, and, immediately, the electrodes were incubated in the anti-cortisol solution of 5 µg/mL concentration for 90 min in the fridge at 4 °C (Figure 2d). Finally, to deactivate the remaining carboxylic acid groups, the electrodes were rinsed with PBS, then incubated in an ethanolamine solution (1% in PBS) for 30 min.

To detect cortisol, the functionalized electrodes were incubated in a sequence of cortisol solutions with ascending concentrations (5, 10, 15, and 20 ng/mL) for 30 min at 4 °C to ensure antigen conservation (Figure 2e). Each incubation was followed by rigorous rinsing with PBS. Then the printed platform was submerged in ferro/ferricyanide to carry the electrochemical impedance spectroscopy (EIS) for at least 5 min to avoid absorptions and missed readings (until obtaining 5 responses with no significant changes), capturing its response for the corresponding concentration. This was followed by the next concentration and repeating the mentioned steps until the last and highest concentration. This additional irreversible method starts with the lower concentration (5 ng/mL) and ends with the higher concentration (20 ng/mL). This supposes that WEs are single-use and can be used once to detect a cortisol quantity due to the binding of the cortisol and anti-cortisol molecules.

### 2.6. Measuring Cortisol Antigen Concentration

The optimization for the antigen antibody detection was carried out in previous works [19,21,32]. Cortisol concentration was measured electrochemically using EIS. Following the incubation of cortisol in standard solution (PBS), and artificial saliva and human saliva with different concentrations (5, 10, 15, and 20 ng/mL), the microelectrodes were measured in ferro/ferricyanide 10 mM with the following parameters: equilibration time 0 s, fixed potential scan with Edc = 0.154 V and Eac = 0.01 V, frequency range is 0.7 and 1000 Hz over a 5 min period. The sweep parameters allow seeing the behavior of the semi-circle with minimum current going through the electrodes. The resulting semi-circle, also known as a Nyquist plot, was then fitted using Randles equivalent circuit model [32], with the following elements: Rs (solution resistance), Rp (charge transfer resistance), and CPE (constant phase element), an equivalent model of double-layer capacitance [33]. The fitting was performed using ZView v3.3f. Calculation errors of the immunosensor were obtained through quadruplicated repetitions, which was possible thanks to the design of the printed platform, and also comparing the results of other printed platforms using the same conditions.

### 2.7. Human Saliva Sampling Procedure

Five healthy volunteers donated their saliva with Salivette^®^ (Art. No. 51.1534.500, Sarstedt, Germany) sampling device; Salivette^®^ is a device aimed at cortisol determination in saliva. The volunteers were asked to move the swab in their oral cavity for 2 min at a self-selected pace. Then, saliva recovery was achieved by centrifugation at 7000 rpm for 5 min at 4 °C. First, all samples were mixed together to create a pooled saliva sample, which was then used to prepare samples for the calibration curve with spikes of cortisol antigen, similarly to what we documented in Section 2.2.

## 3. Results and Discussion

### 3.1. Characterization of the Functionalized Gold Surface

**Figure 3:**
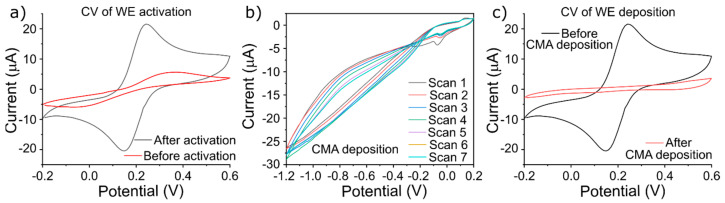
CV of ferro/ferricyanide redox cyclovoltammograms of (**a**) before and after activation of Wes, (**b**) CMA electrodeposition, and (**c**) before and after CMA electrodeposition.

The fully printed electrode on paper (Figure 1c) used for cortisol detection was previously reported by our group [30]. This printed platform was made to fit in a prototype methacrylate chamber consisting of eight rounded–pointed spring-loads to contact with printed PADS and a 10 mL reservoir for electrochemical reaction. This chamber allows us to change printed platforms quickly and use reduced volumes of samples to emulate testing for real-world needs. It will also enable inserting external electrodes as reference and counter electrodes. Incubation and experiments were performed using a 10 mL solution. This printed platform allows several arrangements to obtain valuable statistics sets of experiments. Figure 3a shows the resulting cyclovoltamogram of ferro/ferricyanide on bare Au electrodes before and after activation. Prior to activation, the oxido-reduction peaks of ferro/ferricyanide were barely visible, each showing a peak reaching approximately ±5 µA, after activation, the oxido-reduction peaks of ferro/ferricyanide nearly quadrupled to ±20 µA, thus proving the necessity for the activation step in ensuring the conductivity of the printed electrodes.

To ensure the proper functioning of anti-cortisol antibodies, an intermediate molecule is required to provide support and the proper orientation for its immobilization on the bare Au WE. This is achieved through CMA molecules. As such, the second step after activation is the electrodeposition of CMA molecules onto the Au WEs, as shown in Figure 3b. The parameters of the electrodeposition are as follows: the scan rate was at 25 mV/s, and the switching potential was scanned between −1.2 to 0.2 V for seven cycles.

Figure 3c shows the resulting cyclovoltamogram of ferro/ferricyanide for Au WE before and after CMA deposition. Following the CMA electrodeposition, the oxido-reduction peaks have vanished due to the CMA layer blocking the electron transfer to the ferro/ferricyanide.

The third step of functionalization is the immobilization of the anti-cortisol antibodies on the carboxylic acid groups of the electrodeposited CMA molecules through EDC/NHS reaction, as described in Section 2.5. This step confers on the biosensor its sensitivity and selectivity toward our target antigen.

### 3.2. CMA Electrodeposition Optimization

Given the intrinsic nature of the printed electrodes (nanoparticles structures, non-planar structure, roughness, and more, shown in Figure S2), CMA electrodeposition does not follow standard protocol and must be optimized. A fully blocking layer of CMA on the Au WE ensures a higher number of antibodies linked to the WE, as such, higher sensitivity. The best measure of a CMA deposition is the creation of a fully blocking layer onto the WE, where the ferro/ferricyanide oxido-reduction peaks have disappeared. This is caused by the weak electron transfer kinetics between the CMA layer on the WE and ferro/ferricyanide in the solution.

To obtain a planar response in ferro/ferricyanide after CMA deposition, a series of tests were run where a constant solution of 5 mM CMA was electrodeposited on a range of CV parameters, such as the speed of electrodeposition of 25, 50, and 80 mV/s and the number of cycles (5, 6, and 7). Figure S3 shows the resulting electrodeposition cyclovoltamograms. A key feature of the electrodeposition of diazonium is the formation of an irreversible cathodic wave, where the peak of each cyclovoltamogram is lower after each consecutive cycle. This is caused by the linking of CMA directly onto the gold surface of the printed WEs.

Finally, the resulting electrodeposition was assessed by measuring the oxido-reduction of ferro/ferricyanide with CV; the results can be found in Figure S2. The optimal electrodeposition parameters are seven cycles at 25 mV/s from −1.2 to 0.2 V, which showed a planar cyclovoltamogram without oxido-reduction peaks and capacitive charge; these parameters were then used for all consecutive CMA electrodepositions.

### 3.3. Cortisol’s Detection and Interferences in Standard Solution

**Figure 4:**
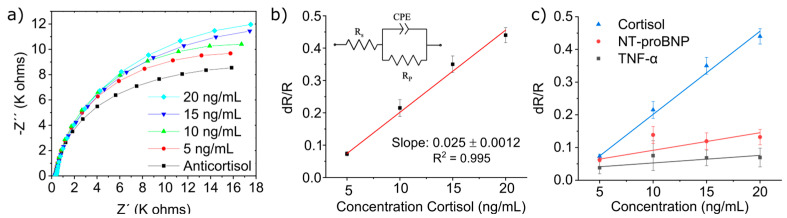
(**a**) Nyquist impedance plots (at standard solution) of cortisol solution analyzed by EIS, (**b**) linearity curve for normalized data obtained from EIS studies for different cortisol concentration in artificial saliva, inset: Randles equivalent circuit model, and (**c**) linearity curve for normalized data obtained from EIS of interference study.

The results of the detection protocol described in Section 2.6 are shown in Figure 4a, which shows the Nyquist plot (an EIS measurement presentation) for cortisol detection on electrode *n* = 4. After antibody immobilization, the electrode was measured by EIS, from which we obtained the first semi-circle. This was followed by incubating the biofunctionalized electrode in 5 ng/mL of cortisol, which, when measured, provided us with a larger semi-circle. This increase in impedance is the result of the binding of the cortisol to its anti-cortisol antibody on the electrode’s surface, which infers correct recognition of cortisol in the solution. Successive increase in cortisol concentrations has yielded larger and larger Nyquist plots, thus indicating an excellent response to cortisol concentration. The reason that the semi-circle increases is that by binding to the anti-cortisol antibody, the cortisol decreases the kinetics of electron transfer to the ferro/ferricyanide solution, thus increasing the system’s impedance.

To study the sensor’s sensitivity to cortisol, the Nyquist plot semi-circles were fitted with Randles equivalent circuit model (Figure 4b inset). This is the most common model for EIS in the literature that represents the behavior seen in the measurement (the resulting semi-circles) [34]. When applied, it has an error rate of <1%.

The Nyquist plot is represented in two vectors, the imaginary part of the impedance Z″, which is a function of the phase shift caused by the alternative current used in the measurement, and the real part of impedance Z′, which increased as a function of cortisol concentration. By fitting the Nyquist plot with Randles equivalent circuit, we can then ascribe the increase in the real impedance Z′ to the increase in the element Rp, which mathematically represents the resistance between the WE and the electrolyte. The bonding of cortisol due to increasing its concentration decreases the electrode transfer kinetics of the WE. Therefore, it increases Rp of the WE. This means that Rp correlates well with the concentration of cortisol being measured. To normalize the data between various electrodes, as shown in Figure 4b, we followed the simple equation of ΔR/R (where ΔR = Rp (cortisol) − Rp (antibodies)/Rp (antibodies)). The results show that the normalized data were linear to cortisol concentrations in the measured range, indicating a great deal of sensitivity towards cortisol of 25 Ω·ng/mL with a correlation coefficient of 0.995 and a limit of detection (LOD) of 1.18 ng/mL.

Following these results, we switched to studying the selectivity of our biosensor using the following cytokines: TNF-α, the symptom of an infection, and NT-proBNP, which was identified as the gold standard biomarker for HF diagnosis and therapy monitoring.

The reasoning behind these cytokines is their relations to cortisol in several diseases, most notably heart failure, as well as their presence in saliva. Thus, studying their interference with the biosensor can open the possibility of developing a multi-targeted platform for a variety of diseases.

As such, the same protocol was used using a newly fabricated biosensor but, instead of measuring cortisol concentrations, we used the cytokines (NT-proBNP and TNF-α). The resulting response curves show greater sensitivity to cortisol of 25 Ω·ng/mL, than those of NT-proBNP (with a sensitivity of 3 Ω·ng/mL) and TNF-α (with a sensitivity of 1 Ω·ng/mL) as shown in Figure 4c. These results confirm the greater selectivity of our biosensor to detect cortisol when compared with other molecules found in its vicinity.

### 3.4. Detection of Cortisol in Artificial Saliva

**Figure 5:**
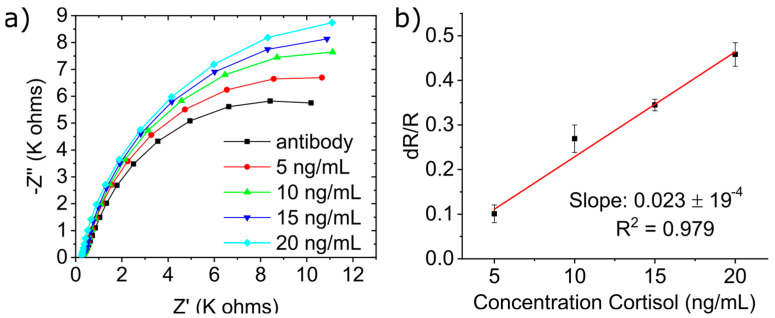
(**a**) Nyquist impedance plots (at artificial saliva) of cortisol solution analyzed by EIS, (**b**) linearity curve for normalized data obtained from EIS studies for different cortisol concentration in artificial saliva.

Section 2.2 describes the synthesis of artificial saliva (A.S). A.S’s main purpose is to simulate the complexity of real saliva matrix. It was used in the same manner as previous sections (Section 3.3) with the alteration that, instead of a standard solution, the cortisol was dissolved in A.S. Cortisol solutions in A.S were prepared with varying concentrations concentration (5, 10, 15, and 20 ng/mL). The results in Figure 5a show that the biosensor has excellent responsiveness to the concentration of cortisol prepared with the A.S as the Nyquist plots increased with the increase in cortisol concentration.

The normalized data, plotted in Figure 5b, yielded a sensitivity of 23 Ω·ng/mL with a correlation coefficient of 0.979 and LOD of 1.09 ng/mL. These results are comparable to the ones obtained with the standard solution, therefore, proving that the biosensor is able to detect cortisol within a complex matrix.

Matrix effect is a phenomenon observed when working with biological fluids. It is characterized by either a loss in response (ion suppression) or an increase in response (ion enhancement) of the devices when present. This is due to the alteration of the ionization efficiency of target analytes in the presence of co-eluting compounds within the same matrix. Matrix effects can be observed [35]. This renders the detection of said analytes completely useless as it alters the response and, subsequently, its measured concentration [36]. Thankfully, the small variability shown in our biosensing platform is an indication of its ability to detect cortisol even in a complex matrix.

### 3.5. Detection of Cortisol in Human Saliva

**Figure 6:**
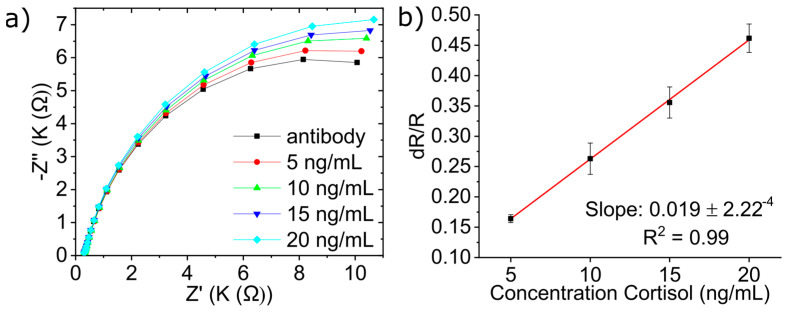
(**a**) Nyquist impedance plots (at real saliva) of cortisol solution analyzed by EIS, (**b**) linearity curve for normalized data obtained from EIS studies for different cortisol concentrations in real saliva.

The third stage of the biosensor development is to test the biosensor with spiked human saliva. As mentioned, the protocol of the collected saliva can be found in Section 2.6. This was performed to study nonspecific bonding in a physiological medium. The same procedure was performed for each concentration as with the previous experiments. The EIS measurements are shown in Figure 6a. As expected, the biosensor showed great responsiveness to the concentration of cortisol in the spiked human saliva; this is shown by the increase in the Nyquist plot in response to the increased concentration of cortisol. The normalized data for human saliva, shown in Figure 6b, yielded a sensitivity of 19 Ω·ng/mL with a correlation coefficient of 0.99 and LOD of 0.81 ng/mL. The remarkable comparativeness of the results demonstrates the feasibility of using the biosensor to detect cortisol in human saliva samples.

The slight reduction in sensitivity between matrices can be attributed to the matrix effect of A.S and human saliva on the cortisol detection. The increased value of the normalized data can be explained due to the presence of cortisol in the saliva that, in this trial, is used as blank.

### 3.6. Cortisol Quantification in Saliva Samples by Standard Addition Method (SAM)

To simulate the saliva sample analysis with an unknown cortisol concentration, three aliquots (450 µL) of the pooled saliva sample (PSS) were spiked with different volumes of 100 ng mL^−1^ cortisol standard solution, obtaining a final concentration of 3, 6, and 10 ng mL^−1^ of cortisol and then named “unknown sample” I, II, and III. SAM was carried out by first preparing SAM samples where a constant volume (50 µL) of the “unknown sample” was added to each of a quartet of 1.5 mL Eppendorf Lo-bind centrifuge tubes (Eppendorf, France). A total of 950 µL of PBS was added to the first tube to obtain the sample C0. Then, an increasing volume of the 100 ng mL^−1^ cortisol standard solution was added to each subsequent tube, before rounding the solutions in each tube to 1 mL with PBS, thus obtaining three samples, C1, C2, and C3, with a known SAM concentrations, where they were analyzed by EIS.

Data obtained from SAM analysis are summarized in Table 1. The precision of our method was calculated by estimating the difference between the cortisol concentrations determined using SAM and their expected values. Based on these results, our biosensor shows good precision to determine the unknown concentration of cortisol in real saliva. These results confirm that our biosensor is highly sensitive to the variation in the unknown concentration of cortisol.

When comparing our device with selected cortisol antibody-based electrochemical sensors in the literature, summarized in Table 2, our proposed biosensing platform shows a comparable LOD to most of the reported works. This demonstrates the potential of the present work in comparison with the current literature while also offering a novel fabrication method (inkjet printing technology) that includes the intrinsic properties of a paper-based device (low-cost, pump-free microfluidic, and eco-friendly, among others). Moreover, our device allows us to repeat the measurement of the sample four times, thus, setting us up to multiplex it for four different analytes in the future, thus demonstrating the biosensing platform’s applicability.

## 4. Conclusions

The development of a new biosensor platform for the detection of cortisol in human saliva samples by EIS is presented in this study. This is the first biosensor platform that is based on inkjet-printed Au electrodes on a paper substrate, and bio-functionalization is optimized for this rough additive technology. The biosensing platform was bio-functionalized by CMA electrodeposition and anti-cortisol antibodies immobilization, resulting in a sensitive device with a good performance and sensitivity for detecting cortisol, even in the presence of other potential interferences such as NT-proBNP and TNF-α. Tests performed in both artificial and human saliva demonstrated the biosensing platform’s capability to detect cortisol in physiological ranges, with a linear relationship between increased cortisol concentration and charge transfer resistance. The biosensing platform shows very promising results also in analyzing artificial saliva and human saliva. Furthermore, the biosensing printed platform allows for further implementation of a multi-parametric biosensing tool for diagnosis and/or monitoring of related diseases using saliva samples.

## Figures and Tables

**Table 1:** Data obtained from the analysis of three saliva samples (corresponding to three aliquots of PSS spiked with different cortisol) by SAM.

Sample Name	Added Concentration (ng/mL)	Dilution Factor	Calculated Concentration *	Bias
Sample I	3	19.1	2.8 ± 0.2 ng mL^−1^ (CV = 7%)	6%
Sample II	6	19.8	5.7 ± 0.2 ng mL^−1^ (CV = 3%)	5%
Sample III	10	19.2	10.2 ± 0.5 ng mL^−1^ (CV = 5%)	2%

* Calculated cortisol concentrations determined using SAM.

**Table 2:** Comparison of different cortisol electrochemical detection.

Technique	Electrode	Immobilizing Biomolecules	Analyte	Linear Range (ng mL^−1^)	Limit of Detection (LOD) (ng mL^−1^)	References
EIS	-	Anti-cortisol antibody	Human Tears	0.05–200	21.66	[37]
SWV *	Graphite	Anti-cortisol antibody	Human saliva	0.5–55.5	1.7	[38]
Amperometry	Reduced graphene oxide	Anti-cortisol antibody	Human saliva and sweat	0.1–200	0.1	[39]
EIS	Au	Anti-cortisol antibody + BSA	Fish plasma	1440–2170	2750	[40]
EIS	Palladium + MoS_2_	Anti-cortisol antibody	Human sweat	1–500	1	[41]
EIS/capacitance	HfO_2_	Anti-cortisol antibody	Human saliva	2−50	0.66	[27]
EIS	E-beam evaporated Au	Anti-cortisol antibody	Human saliva	1–40	1	[42]
EIS	Inject Printed Au	Anti-cortisol antibody	Standard solution	5–20	1.18	This work
EIS	Inject Printed Au	Anti-cortisol antibody	Artificial Saliva	5–20	1.09	This work
EIS	Inject Printed Au	Anti-cortisol antibody	Human Saliva	5–20	0.81	This work

* Square wave voltammetry.

## Data Availability

The original contributions presented in the study are included in the article/Supplementary Materials, further inquiries can be directed to the corresponding author.

## Supplementary Materials

The following supporting information can be downloaded at: https://www.mdpi.com/article/10.3390/mi15101252/s1, Figure S1: CV in HCl 0.1 M at a scan rate of 20 mV/s applied for the chlorinated process of the printed Ag electrode on paper; Figure S2: Images of Au printed microelectrode (a) digital microscope image and (b,c) SEM microscope; Figure S3: Cyclic voltammograms of CMA electrodeposition onto gold WEs. Potential was scanned from 0.2 V to −1.2 V; 5, 6, and 7 cycles were performed at different scan rates (a) 25 mV/s, (b) 50 mV/s, and (c) 80 mV/s. Cyclic voltammograms of activated WEs before CMA deposition and after 5, 6, and 7 scans of CMA deposition varying scan rate speed (d) 25 mV/s, (e) 50 mV/s, and (f) 80 mV/s.

## References

[1] Solhi E., Hasanzadeh M., Babaie P. (2020). Electrochemical Paper-Based Analytical Devices (ePADs) toward Biosensing: Recent Advances and Challenges in Bioanalysis. Anal. Methods.

[2] Fu L.-M., Wang Y.-N. (2018). Detection Methods and Applications of Microfluidic Paper-Based Analytical Devices. TrAC Trends Anal. Chem..

[3] Adkins J., Boehle K., Henry C. (2015). Electrochemical Paper-Based Microfluidic Devices: Microfluidics and Miniaturization. Electrophoresis.

[4] Zeng F., Duan W., Zhu B., Mu T., Zhu L., Guo J., Ma X. (2019). Paper-Based Versatile Surface-Enhanced Raman Spectroscopy Chip with Smartphone-Based Raman Analyzer for Point-of-Care Application. Anal. Chem..

[5] Ruiz-Vega G., Kitsara M., Pellitero M.A., Baldrich E., del Campo F.J. (2017). Electrochemical Lateral Flow Devices: Towards Rapid Immunomagnetic Assays. ChemElectroChem.

[6] Castillo Leon J. (2014). Lab-on-a-Chip Devices and Micro-Total Analysis Systems.

[7] Paschoalino W.J., Kogikoski S., Barragan J.T.C., Giarola J.F., Cantelli L., Rabelo T.M., Pessanha T.M., Kubota L.T. (2019). Emerging Considerations for the Future Development of Electrochemical Paper-Based Analytical Devices. ChemElectroChem.

[8] Hogenelst K., Soeter M., Kallen V. (2019). Ambulatory Measurement of Cortisol: Where Do We Stand, and Which Way to Follow?. Sens. Bio-Sens. Res..

[9] Sun L.-J., Xie Y., Yan Y.-F., Yang H., Gu H.-Y., Bao N. (2017). Paper-Based Analytical Devices for Direct Electrochemical Detection of Free IAA and SA in Plant Samples with the Weight of Several Milligrams. Sens. Actuators B Chem..

[10] e Silva R.F., Longo Cesar Paixão T.R., Der Torossian Torres M., de Araujo W.R. (2020). Simple and Inexpensive Electrochemical Paper-Based Analytical Device for Sensitive Detection of Pseudomonas Aeruginosa. Sens. Actuators B Chem..

[11] Zheng W., Wang K., Xu H., Zheng C., Cao B., Qin Q., Jin Q., Cui D. (2021). Strategies for the Detection of Target Analytes Using Microfluidic Paper-Based Analytical Devices. Anal. Bioanal. Chem..

[12] Moya A., Sowade E., del Campo F.J., Mitra K.Y., Ramon E., Villa R., Baumann R.R., Gabriel G. (2016). All-Inkjet-Printed Dissolved Oxygen Sensors on Flexible Plastic Substrates. Org. Electron..

[13] Tortorich R., Shamkhalichenar H., Choi J.-W. (2018). Inkjet-Printed and Paper-Based Electrochemical Sensors. Appl. Sci..

[14] Määttänen A., Vanamo U., Ihalainen P., Pulkkinen P., Tenhu H., Bobacka J., Peltonen J. (2013). A Low-Cost Paper-Based Inkjet-Printed Platform for Electrochemical Analyses. Sens. Actuators B Chem..

[15] da Costa T.H., Song E., Tortorich R.P., Choi J.-W. (2015). A Paper-Based Electrochemical Sensor Using Inkjet-Printed Carbon Nanotube Electrodes. ECS J. Solid State Sci. Technol..

[16] Shamkhalichenar H., Choi J.-W. (2017). An Inkjet-Printed Non-Enzymatic Hydrogen Peroxide Sensor on Paper. J. Electrochem. Soc..

[17] Hu C., Bai X., Wang Y., Jin W., Zhang X., Hu S. (2012). Inkjet Printing of Nanoporous Gold Electrode Arrays on Cellulose Membranes for High-Sensitive Paper-Like Electrochemical Oxygen Sensors Using Ionic Liquid Electrolytes. Anal. Chem..

[18] Yamaji M., Tsutamoto T., Kawahara C., Nishiyama K., Yamamoto T., Fujii M., Horie M. (2009). Serum Cortisol as a Useful Predictor of Cardiac Events in Patients With Chronic Heart Failure: The Impact of Oxidative Stress. Circ. Heart Fail..

[19] Ben Halima H., Bellagambi F.G., Brunon F., Alcacer A., Pfeiffer N., Heuberger A., Hangouët M., Zine N., Bausells J., Errachid A. (2022). Immuno Field-Effect Transistor (ImmunoFET) for Detection of Salivary Cortisol Using Potentiometric and Impedance Spectroscopy for Monitoring Heart Failure. Talanta.

[20] Halima H.B., Zine N., Gallardo-Gonzalez J., Aissari A.E., Sigaud M., Alcacer A., Bausells J., Errachid A. A Novel Cortisol Biosensor Based on the Capacitive Structure of Hafnium Oxide: Application for Heart Failure Monitoring. Proceedings of the 2019 20th International Conference on Solid-State Sensors, Actuators and Microsystems & Eurosensors XXXIII (TRANSDUCERS & EUROSENSORS XXXIII).

[21] Ben Halima H., Zine N., Nemeir I.A., Pfeiffer N., Heuberger A., Bausells J., Elaissari A., Jaffrezic-Renault N., Errachid A. (2024). An ImmunoFET Coupled with an Immunomagnetic Preconcentration Technique for the Sensitive EIS Detection of HF Biomarkers. Micromachines.

[22] Zangheri M., Cevenini L., Anfossi L., Baggiani C., Simoni P., Di Nardo F., Roda A. (2015). A Simple and Compact Smartphone Accessory for Quantitative Chemiluminescence-Based Lateral Flow Immunoassay for Salivary Cortisol Detection. Biosens. Bioelectron..

[23] Qin Z., Chan W.C.W., Boulware D.R., Akkin T., Butler E.K., Bischof J.C. (2012). Significantly Improved Analytical Sensitivity of Lateral Flow Immunoassays by Using Thermal Contrast. Angew. Chem. Int. Ed..

[24] Parlak O., Keene S.T., Marais A., Curto V.F., Salleo A. (2018). Molecularly Selective Nanoporous Membrane-Based Wearable Organic Electrochemical Device for Noninvasive Cortisol Sensing. Sci. Adv..

[25] Stefan R.-I., van Staden J., Aboul-Enein H. (2000). Design and Use of Electrochemical Sensors in Enantioselective High Throughput Screening of Drugs. A Minireview. Comb. Chem. High Throughput Screen..

[26] Sekar M., Sriramprabha R., Sekhar P.K., Bhansali S., Ponpandian N., Pandiaraj M., Viswanathan C. (2020). Review—Towards Wearable Sensor Platforms for the Electrochemical Detection of Cortisol. J. Electrochem. Soc..

[27] Ben Halima H., Zine N., Bausells J., Jaffrezic-Renault N., Errachid A. (2022). A Novel Cortisol Immunosensor Based on a Hafnium Oxide/Silicon Structure for Heart Failure Diagnosis. Micromachines.

[28] Zhang Y., Ren T., He J. (2018). Inkjet Printing Enabled Controllable Paper Superhydrophobization and Its Applications. ACS Appl. Mater. Interfaces.

[29] Bellagambi F.G., Baraket A., Longo A., Vatteroni M., Zine N., Bausells J., Fuoco R., Di Francesco F., Salvo P., Karanasiou G.S. (2017). Electrochemical Biosensor Platform for TNF-α Cytokines Detection in Both Artificial and Human Saliva: Heart Failure. Sens. Actuators B Chem..

[30] Zea M., Moya A., Villa R., Gabriel G. (2022). Reliable Paper Surface Treatments for the Development of Inkjet-Printed Electrochemical Sensors. Adv. Mater. Interfaces.

[31] Fischer L.M., Tenje M., Heiskanen A.R., Masuda N., Castillo J., Bentien A., Émneus J., Jakobsen M.H., Boisen A. (2009). Gold Cleaning Methods for Electrochemical Detection Applications. Microelectron. Eng..

[32] Ben Halima H., Bellagambi F.G., Hangouët M., Alcacer A., Pfeiffer N., Heuberger A., Zine N., Bausells J., Elaissari A., Errachid A. (2023). A Novel Electrochemical Strategy for NT-proBNP Detection Using IMFET for Monitoring Heart Failure by Saliva Analysis. Talanta.

[33] Barhoumi L., Bellagambi F., Vivaldi F., Baraket A., Clément Y., Zine N., Ben Ali M., Elaissari A., Errachid A. (2019). Ultrasensitive Immunosensor Array for TNF-α Detection in Artificial Saliva Using Polymer-Coated Magnetic Microparticles onto Screen-Printed Gold Electrode. Sensors.

[34] Ben Halima H., Bellagambi F.G., Alcacer A., Pfeiffer N., Heuberger A., Hangouët M., Zine N., Bausells J., Elaissari A., Errachid A. (2021). A Silicon Nitride ISFET Based Immunosensor for Tumor Necrosis Factor-Alpha Detection in Saliva. A Promising Tool for Heart Failure Monitoring. Anal. Chim. Acta.

[35] Zhou W., Yang S., Wang P.G. (2017). Matrix Effects and Application of Matrix Effect Factor. Bioanalysis.

[36] Gosetti F., Mazzucco E., Zampieri D., Gennaro M.C. (2010). Signal Suppression/Enhancement in High-Performance Liquid Chromatography Tandem Mass Spectrometry. J. Chromatogr. A.

[37] Cardinell B.A., Spano M.L., La Belle J.T. (2019). Toward a Label-Free Electrochemical Impedance Immunosensor Design for Quantifying Cortisol in Tears. Crit. Rev. Biomed. Eng..

[38] Kämäräinen S., Mäki M., Tolonen T., Palleschi G., Virtanen V., Micheli L., Sesay A.M. (2018). Disposable Electrochemical Immunosensor for Cortisol Determination in Human Saliva. Talanta.

[39] Tuteja S.K., Ormsby C., Neethirajan S. (2018). Noninvasive Label-Free Detection of Cortisol and Lactate Using Graphene Embedded Screen-Printed Electrode. Nano-Micro Lett..

[40] Pali M., Garvey J.E., Small B., Suni I.I. (2017). Detection of Fish Hormones by Electrochemical Impedance Spectroscopy and Quartz Crystal Microbalance. Sens. Bio-Sens. Res..

[41] Kinnamon D., Ghanta R., Lin K.-C., Muthukumar S., Prasad S. (2017). Portable Biosensor for Monitoring Cortisol in Low-Volume Perspired Human Sweat. Sci. Rep..

[42] Upasham S., Tanak A., Jagannath B., Prasad S. (2018). Development of Ultra-Low Volume, Multi-Bio Fluid, Cortisol Sensing Platform. Sci. Rep..

