# Supplementary material for: Salivary Cortisol Detection with a Fully Inkjet-Printed Paper-Based Electrochemical Sensor
Source: Micromachines (Basel). 2024 Oct 12;15(10):1252. doi: 10.3390/mi15101252 (PMC11509315; doi:10.3390/mi15101252)
Supplement: Supplementary file 1 [file micromachines-15-01252-s001.zip › micromachines-3172544-supplementary.pdf]

micromachines

Supplementary information

Salivary Cortisol Detection with a Fully Inkjet-Printed Paper-Based Electrochemical Sensor

Miguel Zea [1], Hamdi Ben Halima [2,*], Rosa Villa [1,3], Imad Abrao Nemeir [4], Nadia Zine [5], Abdelhamid Errachid [5] and Gemma Gabriel [1,3]

1 Instituto de Microelectrónica de Barcelona IMB-CNM (CSIC), Campus Universitat Autònoma de Barcelona, 08193 Cerdanyola del Vallès, Spain
2 Institut UTINAM, UMR CNRS 6213, Université de Franche-Comté, 16 Route de Gray, 25030 Besançon, France
3 CIBER de Bioingeniería, Biomateriales y Nanomedicina (CIBER-BBN), 08193 Bellaterra, Spain
4 ThEA Group, Department of Chemistry and Biochemistry, Faculty of Arts and Science, Holy Spirit University of Kaslik (USEK), Jounieh P.O. Box 446, Lebanon
5 Institut des Sciences Analytiques (ISA), Université Claude Bernard Lyon 1, 5 Rue de la Doua, 69100 Lyon, France
* Correspondence: hamdi.ben_halima@univ-fcomte.fr

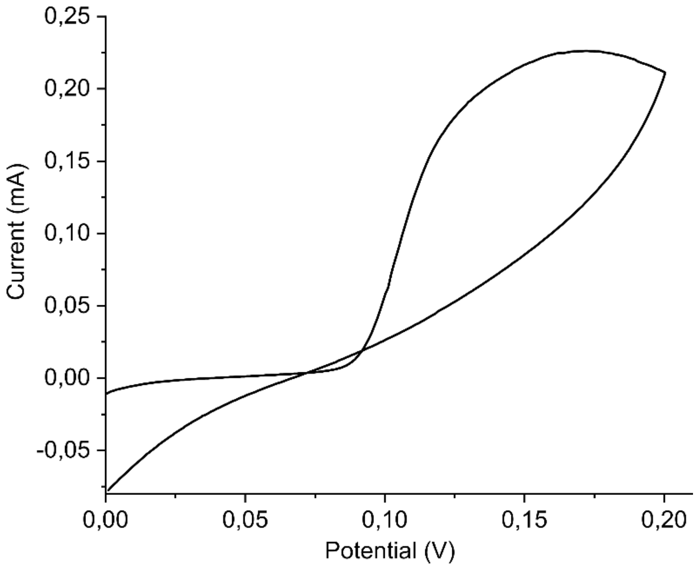

Figure S1. CV in HCl 0.1 M at a scan rate of 20 mV/s applied for the chlorinated process of the printed Ag electrode on paper.

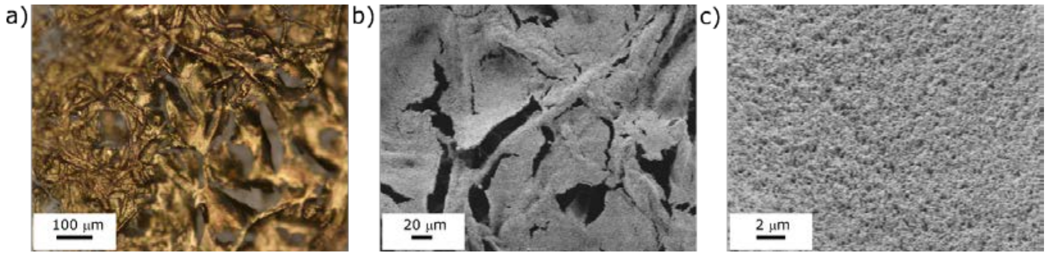

Figure S2. Images of Au printed microelectrode a) digital microscope image and b) and c) SEM microscope.

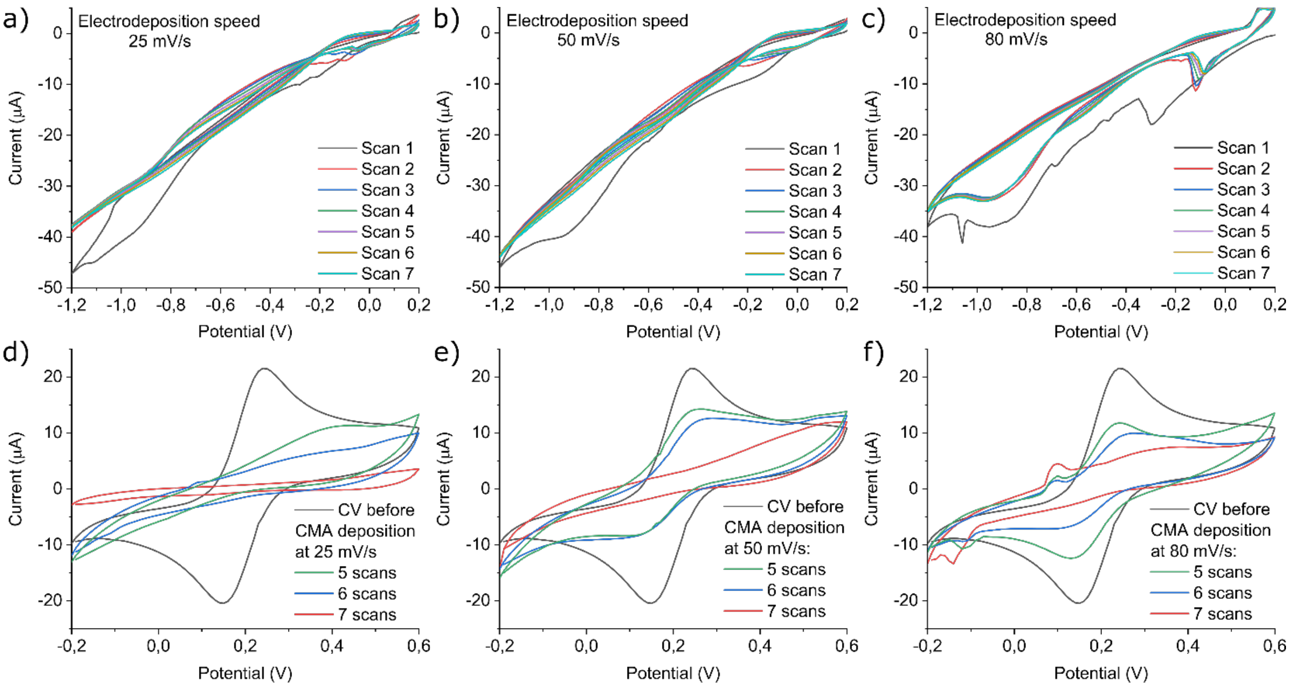

Figure S3. Cyclic voltammograms of CMA electrodeposition onto gold WEs. Potential was scanned from 0.2 V to −1.2 V. 5, 6, and 7 cycles were performed at different scan rates a) 25 mV/s, b) 50 mV/s, and c) 80 mV/s. Cyclic voltammograms of activated WEs before CMA deposition and after 5, 6, and 7 scans of CMA deposition varying scan rate speed d) 25 mV/s, e) 50 mV/s, and f) 80 mV/s.
